# Epidermal-specific deletion of CD44 reveals a function in keratinocytes in response to mechanical stress

**DOI:** 10.1038/cddis.2016.342

**Published:** 2016-11-10

**Authors:** M Shatirishvili, A S Burk, C M Franz, G Pace, T Kastilan, K Breuhahn, E Hinterseer, A Dierich, L Bakiri, E F Wagner, H Ponta, T N Hartmann, M Tanaka, V Orian-Rousseau

**Affiliations:** 1 Karlsruhe Institute of Technology, Institute of Toxicology and Genetics, Karlsruhe, Germany; 2 University of Heidelberg, Institute of Physical Chemistry, Heidelberg, Germany; 3 Karlsruhe Institute of Technology, DFG-Center for Functional Nanostructures, Karlsruhe, Germany; 4 Heidelberg University Hospital, Institute of Pathology, Heidelberg, Germany; 5 Laboratory for Immunological and Molecular Cancer Research, Third Medical Department with Hematology, Medical Oncology, Hemostaseology, Infectious Diseases, and Rheumatology, Oncologic Center, Paracelsus Medical University, Salzburg, Austria; 6 Institut Clinique de la Souris Illkirch, Illkirch, France; 7 Spanish National Cancer Centre, Genes Development and Disease Group, Cancer Cell Biology Programme, Madrid, Spain; 8 Institute for Integrated Cell-Material Sciences (WPI iCeMS), Kyoto University, Kyoto 606-8501, Japan

## Abstract

CD44, a large family of transmembrane glycoproteins, plays decisive roles in physiological and pathological conditions. CD44 isoforms are involved in several signaling pathways essential for life such as growth factor-induced signaling by EGF, HGF or VEGF. CD44 is also the main hyaluronan (HA) receptor and as such is involved in HA-dependent processes. To allow a genetic dissection of CD44 functions in homeostasis and disease, we generated a *Cd44* floxed allele allowing tissue- and time-specific inactivation of all CD44 isoforms *in vivo*. As a proof of principle, we inactivated *Cd44* in the skin epidermis using the *K14Cre* allele. Although the skin of such *Cd44*^*Δker*^ mutants appeared morphologically normal, epidermal stiffness was reduced, wound healing delayed and TPA induced epidermal thickening decreased. These phenotypes might be caused by cell autonomous defects in differentiation and HA production as well as impaired adhesion and migration on HA by *Cd44*^*Δker*^ keratinocytes. These findings support the usefulness of the conditional *Cd44* allele in unraveling essential physiological and pathological functions of CD44 isoforms.

CD44 is a large family of transmembrane glycoproteins controlling cell behavior and cell fate (reviewed in ref. 1). The inclusion of different combinations of variant exons in the CD44 extracellular domain gives rise to a plethora of CD44 variant isoforms with distinct extracellular domains. The smallest CD44 isoform, namely CD44s (standard), is devoid of variant exons (reviewed in ref. 1). CD44 isoforms are expressed in many different tissues, CD44s being the predominant form expressed at early stages of embryogenesis.^2^ Accumulating evidence indicate that CD44 is a master regulator of cell signaling. For example, the v3-containing heparan sulphated CD44 isoforms (CD44v3) bind growth factors such as HB-EGF or b-FGF.^3^ During limb development such a specific heparan sulphated isoform is expressed in cells of the apical ectodermal ridge and is involved in the presentation of FGF to its receptor FGFR, present on the underlying mesenchymal cells.^4^ CD44v3-mediated activation of FGFR leads to limb outgrowth. CD44v6 isoforms collaborate with the receptor tyrosine kinases MET and VEGFR-2 that are crucial during development (reviewed in ref. 1). The extracellular part of CD44v6 binds HGF and VEGF(ref. 5) and is required for activation of the receptors while the cytoplasmic domain mediates signal transduction on binding to Ezrin–Radixin–Moesin proteins that provide a link to the actin cytoskeleton.^6^ In physiological conditions, MET-CD44 association is needed for synaptic transmission in the brainstem respiratory rhythm-generating network,^7^ whereas in patholological conditions, cooperation between CD44v6 and MET is essential for tumor growth and metastasis of pancreatic cancer cells.^8^ More recently, CD44, an established Wnt target gene, was also shown to act as a Wnt co-receptor at the level of LRP6 in a positive feedback loop.^9^

Hyaluronan (HA) is the best characterized ligand of CD44(ref. 10) and CD44/HA interactions control differentiation, proliferation, survival and migration of cells thereby playing a major role in tumor progression and metastasis.^11^ Binding of high molecular weight HA to CD44 augments CXCL12-induced signaling and subsequent angiogenesis^12^ and HA binding to CD44 and subsequent interaction with neural Wiskott–Aldrich syndrome protein induces actin polymerization and EGFR activation.^13^

Despite the participation of CD44 in crucial signaling pathways, conventional *Cd44* germ-line knockout mice (*Cd44*^*−/−*^) develop normally and only display mild phenotypes in adulthood.^14, 15^ The possibility that a substitute molecule takes over the co-receptor function of CD44 in the *Cd44*^*−/−*^ mice was therefore proposed. Several lines of evidence support this hypothesis. First, in clear contradiction with the mild changes observed in the *Cd44*^*−/−*^ mice, transgenic animals expressing an antisense CD44 cDNA under the control of a keratin-5 promoter displayed a drastic skin phenotype.^16^ Second, *Cd44*^*−/−*^*, Met*^*+/−*^ mice are haplo-insufficient and most mutants die briefly after birth from a breathing defect that results from impaired synaptogenesis and axon myelination.^7^ This result suggests that CD44 and MET co-operate *in vivo* and that *Cd44*^*−/−*^ mice establish a rescue function to maintain activity of the MET pathway, which is not sufficient when MET levels are reduced. Such a rescue function is exemplified in hepatocytes where the intercellular cell adhesion molecule ICAM-1 takes over the co-receptor function of CD44 for MET when *Cd44* is inactivated.^17^

For all these reasons, the best way to study CD44 functions *in vivo* is by cell-specific conditional inactivation. Therefore, we engineered a *Cd44* floxed allele which when combined with Cre eliminates an essential constant exon (exon 3 designated c3, Figure 1a) and thereby all CD44 isoforms. Using this novel allele, we first investigated the role of CD44 in the skin. The skin is a protective barrier against influences of the external environment and the tightly controlled proliferation and differentiation of epidermal keratinocytes along the three epidermal layers is essential for homeostasis of this highly regenerative tissue. On wounding, a complex process involving inflammation, proliferation and remodeling^18^ takes place by which basal keratinocytes together with hair follicle stem cells are able to replace the epidermal cell population.^19, 20^ Deregulation of these processes can lead to severe skin diseases such as hyperkeratosis, hyperplasia or cancer.

In this paper we show that while the skin of keratinocyte-deficient *Cd44 mice* (*Cd44*^*Δker*^) appears normal, epidermal stiffness is decreased and *in vivo* wound healing delayed. Moreover, 12-O-tetradecanoylphorbol-13-acetate (TPA)-induced cell proliferation and differentiation is also disturbed. These defects are cell autonomous and *Cd44*^*Δker*^ keratinocytes display a decreased production of HA, impaired adhesion, morphological dynamics and locomotion.

## Results

### Consequence of targeted *Cd44* deletion in K14 expressing cells on skin homeostasis

**Figure 1:**
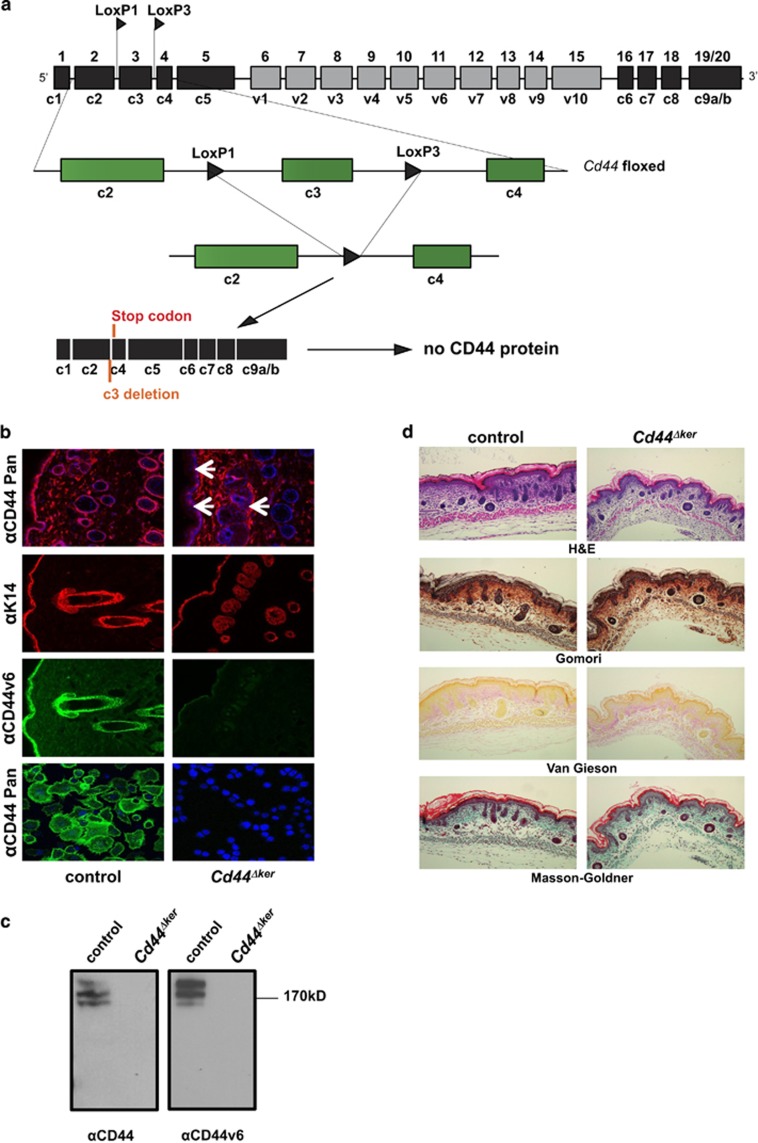
Knockout of CD44 in the skin. (**a**) Schematic representation of *Cd44* conditional knockout allele. Upon crossing of the *Cd44* floxed mice with *K14*Cre mice deletion of the *Cd44* exon 3 leads to a frameshift and a stop codon disrupting the expression of all isoforms. (**b**) A pan CD44 antibody (IM7; red, green), a CD44v6 specific antibody (9A4; green) or a keratin 14 antibody (red) was used for indirect immune fluorescence staining of skin sections or keratinocytes (lower panel) harvested from *Cd44*^*Δker*^ mice or control animals. Nuclei were counterstained with DAPI (blue). White arrows indicate areas where CD44 is removed (epidermis, hair follicles). (**c**) Western Blot analysis of lysates of keratinocytes, obtained from *CD44*^*Δker*^ *Cd44* and control animals using a pan CD44 antibody (KM201) or a CD44v6 specific antibody (*α*9A4). (**d**) New born *Cd44*^*Δker*^ or control skin stained for: H&E, Gomori Trichrome, Verhoeff‘s Van Gieson and Masson Goldner

A *Cd44* floxed allele was generated by gene targeting in ES cells. Exon 3, present in all CD44 isoforms, was flanked by LoxP sites. Cre recombinase leads to excision of exon 3, to the generation of an in-frame stop codon and subsequent disruption of protein synthesis in the cells where Cre is expressed leading to loss of all CD44 isoforms (Figure 1a). After germ-line transmission of the *Cd44* floxed allele, *Cd44* floxed mice were crossed with *K14*Cre mice^21^ to generate *Cd44*^*Δker*^ animals. The *K14*Cre transgene is expressed as early as 9.5 days post-coitum in complex epithelia such as the epidermis.^21^ The removal of *Cd44* in the whole epidermis and in isolated primary keratinocytes of *Cd44*^*Δker*^ mice was confirmed by histological stainigs (1b) PCR, Southern blot and Western blot analyses while qRT-PCR revealed a 70% decrease in CD44 mRNA (Figures 1b, c and Supplementary Figure S1). The corresponding mRNA is not expressed as protein as shown in the western blot (Figure 1c). The western blot analysis also reveals that the majority of CD44 proteins expressed in the epidermis of control mice contain the v6 exon since the staining pattern with CD44v6 specific antibodies is nearly identical to that obtained with a pan CD44 antibody that recognizes all CD44 isoforms (Figure 1c). We confirmed that *Cd44* was also inactivated in other *K14*-expressing organs such as the oral epithelium, tongue, mammary epithelium and thymus (data not shown).

To analyze potential morphological changes in the skin, *Cd44*^*Δker*^ mice and control littermates were killed at different ages and the skin harvested for histological analysis (Figure 1d). A hematoxylin-eosin (H&E) staining, a Gomori staining showing the distribution of the reticulin fibers, a Verhoeff‘s Van Gieson staining detecting collagen fibers and a Masson Goldner staining visualizing connective tissue were performed. As shown in Figure 1d (new born) but also in 2, 6 and 30 weeks old mice, (data not shown) no structural changes could be detected in the skin of *Cd44*^*Δker*^ mice. Specifically, no edema was observed and the number or morphology of the dermal fibroblasts did not significantly differ. Moreover, fibers orientation seemed not to be affected and dermal stability was not disturbed. Finally, no epidermal hyperplasia or atypical keratinocytes were identified. Therefore, no gross abnormalities were observed in the dermis or the epidermis. Observation of *Cd44*^*Δker*^ mice for a long time period (a year) did not reveal any spontaneous skin phenotype (data not shown).

### Lack of CD44 in the skin interferes with wound healing

**Figure 2:**
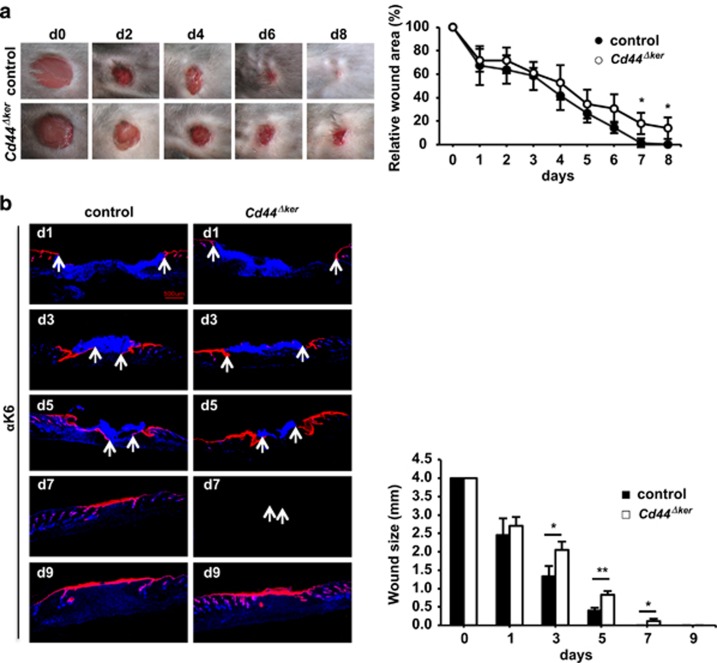
Impaired wound healing in *Cd44*^*Δker*^ mice. (**a**) 4 mm full-thickness excisional dorsal skin wounds were performed on *Cd44*^*Δker*^ and control animals and photographed at the indicated days. Wound closure was quantified by ImageJ and expressed relative to the initial wound area. (**b**) Wound sections were stained for keratin 6 (red) and nuclei counterstained with DAPI (blue). Images were taken by fluorescence microscopy (KEYENCY BZ-9000) using a × 2 objective. Bar=500 *μ*m. Quantification of the distance between the keratinocyte migration fronts (white arrows). Bars represent means ±S.E.M., *n*=6. Student's *t* test: *P<0.05, **P<0.005. Experiments were performed at least three times and gave similar results

We next addressed the response of the skin to stress and performed wound healing experiments in *Cd44*^*Δker*^ mice. To this end 4 mm full-thickness excisional wounds were performed on 10–12-week-old *Cd44*^*Δker*^ mice and control littermates (Figure 2a) and the size of the wound determined. A difference in the relative wound area was observed between the two cohorts from day 1 on. The most drastic and statistically significant difference was observed at day 8, where *Cd44*^*Δker*^ mice showed a striking delay in wound closure while the wounds of *Cd44*-proficient mice were already completely closed (Figure 2a).

To analyze the contribution of keratinocytes in the re-epithelialization (Figure 2b), wound sections were stained for keratin 6 (K6), which is specifically induced in hyper-proliferative and migrating keratinocytes during wound healing.^22, 23, 24^ At each time point the distance between the migration fronts of the keratinocytes was larger in *Cd44*^*Δker*^ mice, further demonstrating that inactivation of *Cd44* in the epidermis leads to delayed wound healing (Figure 2b). In striking contrast, we did not observe any difference in wound closure between the *Cd44*^*−/−*^ germ-line knockout and control mice (Supplementary Figure S2A). This observation confirms that functions of CD44 are substituted in the germ-line knockout keratinocytes as was also the case in hepatocytes.^17^ This substitution apparently does not occur if the inactivation of the *Cd44* gene arises at later times in embryogenesis, as is the case in the conditional knockout (the K14 promoter for Cre expression is active only from E 9,5) mice.

### Impaired keratinocyte proliferation and differentiation in *Cd44*^*Δker*^ mice

**Figure 3:**
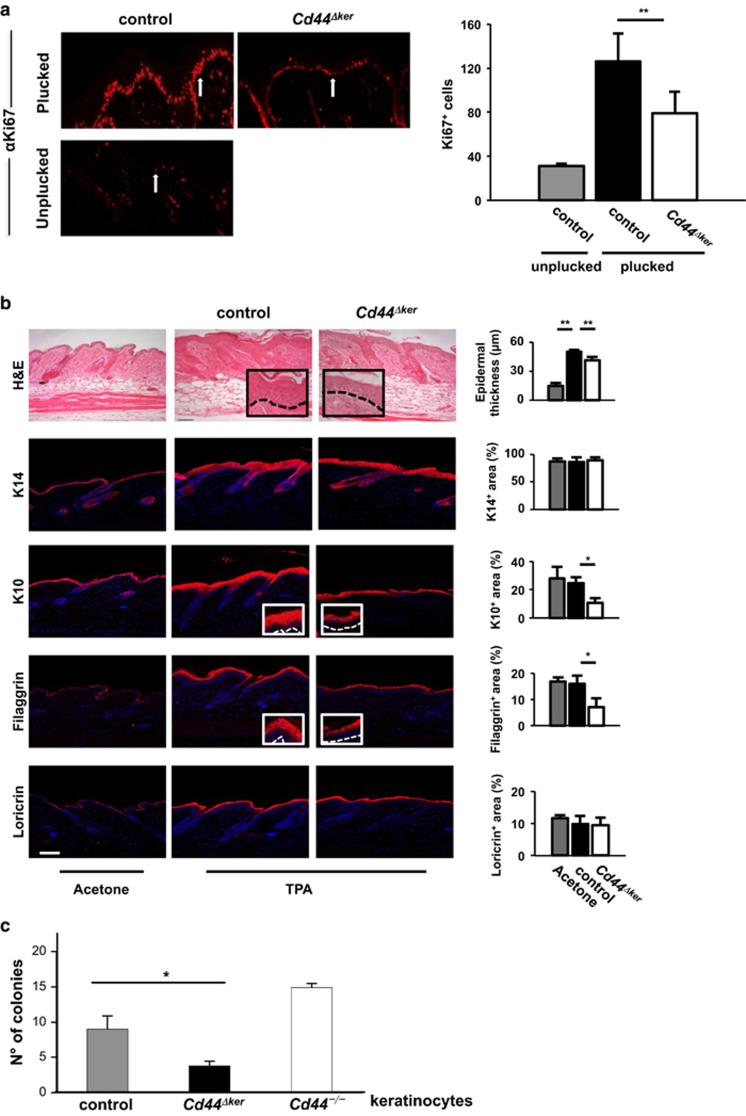
Loss of CD44 leads to impaired keratinocyte proliferation/differentiation. (**a**) Hair plucking was performed on the dorsal skin of *Cd44*^*Δker*^ and control animals. Unplucked skin served as a control. The sections were stained for Ki67 (red) proliferation marker. Quantification is presented on the right side. (**b**) TPA-treated skin sections of *Cd44*^*Δker*^ mice and control animals, and acetone-treated skin sections of control animals were analyzed by H&E (bar=50 *μ*m), K14, K10, filaggrin or loricrin stainings (bar=75 *μ*m). Epidermal/dermal junction is indicated by dashed lines. Quantification of epidermal thickness and of the stained area size, relative to epidermal thickness. (**c**) *Cd44*^*Δker*^, control and germ-line KO keratinocytes were isolated and subjected to colony formation assay. Bars represent means ±S.E.M., *n*=5. Student's *t* test: *P<0.05, **P<0.005. Experiments were performed at least three times and gave similar results

We next tested the response of epidermal *Cd44*^*Δker*^ keratinocytes to hair plucking and to TPA treatment^25^ (Figure 3).

As reported,^26^ hair plucking induced keratinocytes proliferation. Strikingly, quantification of Ki67 staining revealed that the proliferative response of *Cd44*^*Δker*^ keratinocytes was much less pronounced than in controls (Figure 3a). Interestingly, however, hair regrowth did not appear delayed in *Cd44*^*Δker*^ mice (not shown).

TPA treatment induced epidermal thickening of the skin as compared with acetone treatment as measured by H&E staining (Figure 3b). This increase was much less pronounced in *Cd44*^*Δker*^ mice (Figure 3b). Taken together, these results indicate an impaired proliferative response of *Cd44*^*Δker*^ keratinocytes. In support of this, isolated *Cd44*^*Δker*^ keratinocytes showed reduced clonogenic efficiency as compared with controls and germ-line knockout keratinocytes (Figure 3c).

The epidermis consists of four different layers expressing various sets of keratins: The basal layer is characterized by expression of K5 and K14, the spinous layer by expression of K1 and K10,^22, 27^ the granular layer produces filaggrin,^28, 29^ and the cornified layer, loricrin.^30, 31^ We made use of these markers to analyze the impact of CD44 on epidermal differentiation. Skin sections of TPA treated animals were stained immune fluorescently with the markers K14, K10, filaggrin and loricrin (Figure 3b). Fluorescence intensity increased for all markers in TPA treated animals consistent with the induction of epidermal marker expression by TPA. When calculating the ratio of the stained layers relative to the total epidermal thickness, we observed that the relative thickness of each epidermal layer was not changed between acetone-treated controls and TPA-treated *Cd44*-proficient animals. However, in *Cd44*^*Δker*^ mice, we observed a reduction in the relative thickness of the K10 and filaggrin-positive layers while the relative thickness of the K14 and loricrin layers appeared unaffected (Figure 3b). In addition, overall fluorescence intensity appeared weaker. These results indicate that genetic inactivation of *Cd44* in the K14-expressing cells has also an impact on differentiation of keratinocytes. The lack of effect on relative K14 or loricrin levels might be due to the timing of the experimental endpoint.

Consistent with the *in vivo* data, we observed a delayed induction of keratin genes *in vitro,* when treating keratinocytes isolated from *Cd44*^*Δker*^ mice for 2 h with TPA. This was most apparent for Keratin 5 and Keratin 14, and to a lesser extent for Keratin 1 (Supplementary Figure S2B upper part). Interestingly, TPA induction of several genes encoding for AP-1 family members that are robustly induced by TPA, was also delayed (Supplementary Figure S2B lower part).

### Removal of *Cd44* decreased the adhesion of keratinocytes to HA and their migration on HA surfaces

**Figure 4:**
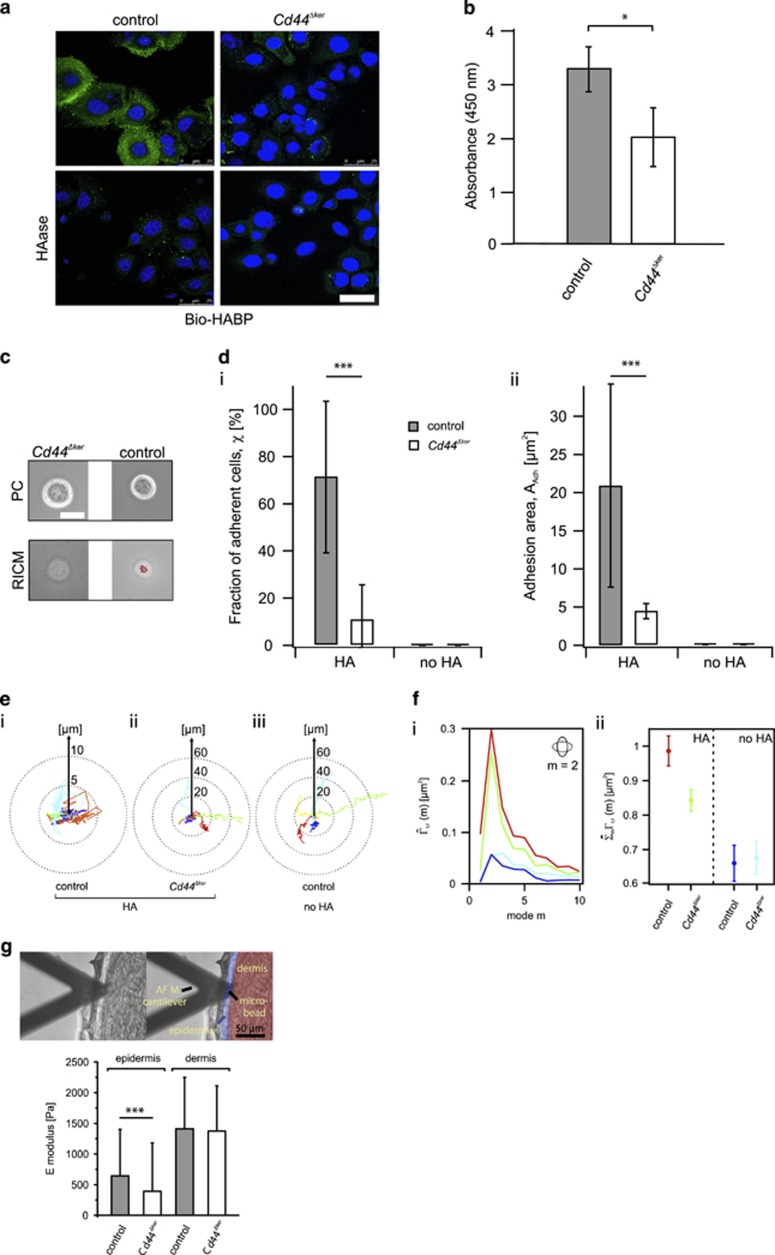
HA production and binding in *CD44*^*Δker*^ keratinocytes. (**a**) HA staining (green) of mouse keratinocytes derived from *Cd44*^*Δker*^ mice or control animals was performed using a Bio-HABP detected with streptavidin-Alexa 488. Nuclei were counterstained with DAPI (blue). Images were taken with a SpE confocal microscope. Bar=25 *μ*m. Control cells were also pretreated with hyaluronidase (HAase) before staining. (**b**) HA production by keratinocytes from *Cd44*^*Δker*^ mice or control animals on incubation for 24 h in culture dishes. Bars ±S.E.M. *n*=3. Student's *t* test: *P<0.05. (**c**) Phase contrast and RICM images of *Cd44*^*Δker*^ and *Cd44*^*+*^ keratinocytes on supported membranes displaying oligo-HA (scheme in Supplementary Figure S3A) at an average intermolecular distance (*<d*_*oligo-HA*_*>)* of ~6 nm at 5 h after cell seeding. Dark spots (red encircled) in RICM images correspond to areas of tight cell adhesion. (**d**) Determination of the fraction of adherent *Cd44*^*Δker*^ and *Cd44*^*+*^ keratinocytes (*χ*) or their adhesion area (*A*_*adh*_) on functionalized (HA) or non-functionalized (no HA) membranes. All data are mean±S.D. of *n*=30 cells of three independent experiments. *P<0.05; **P<0.01; ***P<0.001 (Mann–Whitney test). (**e**) Five exemplary tracks of individual keratinocytes in polar plots obtained from cell tracking of 20 keratinocytes per condition by time-lapse movies. (**f**) Power spectrum of *Cd44*^*Δker*^ and *Cd44*^*+*^ keratinocytes on oligo-HA functionalized membranes or control membranes obtained by a Fourier transformation Ft(*r*(*θ*, *t*)) of the corresponding amplitude maps (Supplementary Figure S3C). This analysis yields the predominant mode *m* of cellular deformation by which keratinocytes dissipated energy. The integration over all modes of deformation, *m*, results in the determination of the total power of deformation 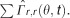 All data are mean±S.E.M. of *n*=20 cells of three independent experiments. (**g**) AFM measurement of full-thickness skin biopsies from new born *Cd44*^*Δker*^ and control mice (pictures). Young's E modulus of mouse epidermal and dermal tissues. AFM measurement of full-thickness skin biopsies from new born Cd44Δker and control mice (pictures). Young's E modulus of mouse epidermal and dermal tissues presented as box-whisker-plots. At least 15 different locations per skin region were tested, using skin slices from at least three different animals. The statistical significance between experimental groups was determined using a one-way ANOVA test: NS=***P<0.001

A prominent component of the extracellular matrix in the skin is HA.^32^ As CD44 is the main receptor for HA^10^ we next performed a HA staining on isolated keratinocytes and on skin sections using the biotinylated HA-binding protein (Bio-HABP). Interestingly, the HABP staining did not reveal changes in the overall HA distribution in the skin sections neither during homeostasis nor during wound healing (Supplementary Figure S3A). This might be due to the contribution of cells from the dermis that still express *Cd44*. In contrast, in the isolated keratinocytes, a positive staining was detected only in *Cd44*^*+*^ keratinocyte cultures as shown in Figure 4a. The decreased HA staining in *Cd44*^*Δker*^ keratinocytes suggests that the cells are either unable to bind to HA and/or to produce HA. We quantified the amount of HA produced by *Cd44*^*+*^ and *Cd44*^*Δker*^ keratinocytes using solid-phase ELISA in cell culture supernatants (Figure 4b). A significant decrease of HA production was observed in the supernatant of *Cd44*^*Δker*^ cultures consistent with previous reports indicating that CD44 directly influences HA production.^33, 34^

We next investigated the adhesion of cells on oligo-HA functionalized membranes (scheme in Supplementary Figure S3B) utilizing a combination of phase contrast and non-invasive live-cell imaging by reflection interference contrast microscopy (RICM). Figure 4c represents phase contrast and corresponding RICM images for *Cd44*^*Δker*^ and *Cd44*^*+*^ (control) keratinocytes. The area of tight cell adhesion can be identified from the dark area in RICM images without any additional fluorescent labeling (Figure 4c, highlighted by a red line).^35^ The fraction of adherent cells (*χ*) was significantly lower for *Cd44*^*Δker*^ cells as compared with *Cd44*^*+*^ keratinocytes (Figure 4d, left). In addition to this defect in the collective cell adhesion, the adhesion area *A*_*adh*_ of individual keratinocytes was also significantly decreased by a factor of 3 on *Cd44* inactivation (Figure 4d, right). These results indicate that the binding ability of *Cd44*^*Δker*^ cells to HA is impaired.

We next investigated the morphological dynamics and migration of *Cd44*^*Δker*^ and *Cd44*^*+*^ keratinocytes. The *Cd44*^*Δker*^ keratinocytes that lack HA binding capability, show much longer and more persistent trajectories on oligo-HA functionalized membranes as compared with *Cd44*-proficient control keratinocytes (Figure 4ei–ii) similarly to control keratinocytes plated on pure membrane (no HA; Figure 4e, iii).

Dynamic morphological fluctuations of cells are active processes due to bending and stretching of their membranes and remodeling of their cytoskeleton by ATP consumption.^36, 37^ The corresponding deformation energy can be estimated from the power spectrum analysis as reported previously.^38^ Figure 4f illustrates power spectra of *Cd44*^*Δker*^ and *Cd44*^*+*^ keratinocytes on oligo-HA functionalized membranes and the corresponding data for *Cd44*^*+*^ cells on pure membranes (no HA; Supplementary Figure S3C). A maximum of the curve at mode *m*=2 suggested that keratinocytes predominantly undergo elliptic deformation as schematically depicted in the inset (Figure 4f left). The summation over all modes of deformation 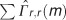 reflecting the total energy consumption was strikingly decreased in *Cd44*^*Δker*^ keratinocytes plated on HA functionalized membranes as compared with control keratinocytes while cells plated on pure membranes (no HA) did not show any deformations (Figure 4f, right).

### Decreased epidermal stiffness in *Cd44*^*Δker*^ mice

One essential role of the skin is to provide elasticity and resistance to stretch^39^ and the HA-related defects of keratinocytes lacking *Cd44* could affect these intrinsic properties of the skin epidermis. We therefore examined the skin mechanical properties using atomic force microscopy (AFM) for nanoindentation, a method that quantifies tissue mechanical and elastic properties.^40^ AFM elasticity measurements were performed on mouse skin tissue slices and the indenter bead was positioned over the dermal or epidermal regions (Figure 4g, shown for new born mice, sections of 2-, 6- and 30-week-old mice gave similar results) in the same experimental set-up.

AFM analysis revealed a striking decrease in epidermal stiffness in *Cd44*^*Δker*^ mice, whereas the dermis was not affected. Reasons for the decrease in epidermal stiffness in *Cd44*^*Δker*^ mice could be reduced HA production and/or lack of CD44-mediated adhesion to HA (Figure 4f). Since the mechanical environment of the wound site is important for the healing process, delayed skin wound healing in *Cd44*^*Δker*^ mice could thus be the collective result of impaired keratinocyte proliferation, CD44/HA-mediated migration and adhesion together with altered biomechanical properties of the epidermis.

## Discussion

CD44 is a multi-tasking family of proteins involved in signaling pathways essential for embryonic development. Therefore the mild phenotype of the *Cd44* germ-line knockout mice^14, 15^ suggested that CD44 is functionally substituted during embryogenesis. Such a lack of phenotype in germ-line knockout mice was described for several other genes and substitutions based on up-regulation of members of the same family or functionally related proteins have been identified. For instance fibroblast growth factor-2 (*Fgf-2*) knockout mice develop normally and display normal liver regeneration.^41^ VEGF was shown to substitute for FGF-2 and the VEGF inhibitor SU5416 inhibited liver regeneration in *Fgf-2* knockout mice but not in control animals.^42^ Similarly, the inactivation of the *tau* gene encoding for a major neuronal microtubule-associated protein essential for neuronal cell morphogenesis and axonal elongation and maintenance only led to mild defects in microtubule stability due to compensation by another protein, MAP-1A.^43^

In the case of CD44 several evidences support substitution events in the *Cd44*^*−/−*^ mouse. For example, the presentation of FGF produced in the apical ectodermal ridge to its receptor FGFR on the underlying mesenchymal cells by CD44v3 during limb development^4^ is overtaken by another heparan sulfate proteoglycan, upregulated in the *Cd44*^*−/−*^ mice.^44^ Furthermore, RHAMM, another HA receptor, compensates for CD44 in the *Cd44*^*−/−*^ mice.^45^ Finally, we have shown that ICAM-1 substitutes for CD44v6 in the *Cd44*^*−/−*^ mice as a co-receptor for MET *in vivo* and *in vitro*.^17^

The most appropriate strategy to identify functions of CD44 *in vivo* in various tissues and different stages of development is the use of a conditional inactivation of the *Cd44* gene with the expectation that a substitute molecule will not be expressed. This strategy additionally allows deciphering cell autonomous functions of CD44 in the context of an otherwise wild-type tissue. Using the Cre-Lox technology to inactivate *Cd44* we show in this paper that specific abrogation of CD44 in the skin epidermis has little apparent impact on the gross morphology of the skin. However, strikingly decreased epidermal stiffness as measured by AFM was detected on removal of *Cd44*. In addition, keratinocytes isolated from *Cd44*^*Δker*^ mice show reduced HA production, impaired adhesion to HA, reduced collective and individual migration, and morphological dynamics on HA functionalized surfaces. These results support a physiological function of CD44 in keratinocytes. Consistently, TPA leads to impaired epidermal thickening and differentiation in *Cd44*^*Δker*^ mice. Delayed TPA response of *Cd44*^*Δker*^ keratinocytes was also observed *in vitro*. Interestingly, we also saw a delayed TPA induction of members of the AP-1 transcription factor, an important regulator of epidermal differentiation.^46, 47, 48^ This suggests a possible modulation of AP-1 expression by *Cd44*. As *Cd44* is itself a well-known target gene of AP-1^(ref^. ^49^^)^ a positive feedback regulation of AP-1 by CD44 might be in place in keratinocytes subjected to differentiation signals.

We also observed a proliferation delay in *Cd44*^*Δker*^ epidermis subjected to hair plucking. In addition, *in vivo* wound healing was delayed in *Cd44*^*Δker*^ animals. Interestingly, this delay was not observed in *Cd44* germ-line knockout mice, further supporting a substitution of CD44 in early embryogenesis.

The most likely explanation for the delayed wound healing in *Cd44*^*Δker*^ mice is an impairment of HA synthesis by keratinocytes and the inability of keratinocytes to attach and migrate on exogenous HA. The importance of HA synthesis for keratinocytes migration has been previously demonstrated by comparison of two different keratinocyte cell lines, carrying the HA synthase 2 (*Has2*) gene in sense or antisense orientation to increase or decrease HA synthesis, respectively.^50^ Keratinocytes expressing the *Has2* sense gene migrated faster in an *in vitro* wounding assay, whereas Has2 antisense cells migrated more slowly. Interestingly, addition of exogenous HA to keratinocyte cultures did not restore the reduced migratory ability of Has2 antisense cells suggesting that the dynamic synthesis of HA controls keratinocyte migration, a key function during wound closure.^50^

The defects described above might result in a change in epidermal stiffness, which in turn affects wound healing. The AFM technology applied to a tissue layer allowed discriminating between the contribution of the dermis and epidermis, and suggests that the decreased epidermal stiffness might prevent rapid wound healing. Indeed, it has been previously demonstrated that stiff surfaces promote wound healing by stimulating epidermal cell proliferation and migration.^51, 52^ *Cd44*^*Δker*^ keratinocytes lack CD44 as the main HA receptor and also produce reduced levels of HA. The absence of CD44-dependent HA binding might thus decrease the stiffness of the wound surface and thereby delay wound healing. Furthermore, the change in the dynamic fluctuation of *Cd44*^*Δker*^ keratinocytes (Figure 4f) might also contribute to the decreased stiffness of the epidermis.

In contrast to our observation, however, one report described delayed wound healing in *Cd44*^*−/−*^ mice due to impaired vascularization.^53^ In another case, decreased HA-mediated keratinocyte differentiation and lipid synthesis that interfered with normal epidermal permeability and barrier function was reported in *Cd44*^*−/−*^ mice.^54, 55^ These contradictory results might be due to the different genetic backgrounds of the mice used in the different experimental settings as reported for genes such as *Egfr*.^56^

The gross morphology of the skin is not altered in *Cd44*^*Δker*^ mice. This is in contradiction with drastic alterations in the structure of the dermis and epidermis of mice in which *Cd44* was abolished in keratinocytes using an antisense CD44 construct expressed under the control of a K5 promoter.^16^ In addition, these *Cd44* knockdown keratinocytes displayed a defect in HA uptake resulting in an accumulation of HA in the dermis. Some of the severe structural changes in the skin of these CD44 antisense mice could be attributed to off-target effects of antisense oligonucleotide expression *in vivo*.^57^ Interestingly, however, a change in skin elasticity, wound repair and keratinocyte proliferation were also observed in these antisense mice consistent with our observations in the *Cd44*^*Δker*^ genetic model.

In conclusion, genetic inactivation of *Cd44* in the keratinocytes allowed to more precisely defining the role of the CD44/HA pair in epidermal repair during wound healing and in the regulation of keratinocyte differentiation, migration and proliferation. We genetically demonstrated important functions of CD44 in skin epidermis in homeostasis and acute stress conditions. Such genetic tools will allow dissecting CD44 functions in skin aging and in pathological situations such as skin hyperplasia and cancer.

## Materials and Methods

### Generation of *Cd44* floxed mice, other mouse strains

pCD44-KO vector consisting of genomic *Cd44* exons 2 and 3 (10,7 kb) derived from the mouse strain 129/01a and a single Lox-P site inserted downstream from exon 3 were obtained from Therese Touchy (Beaverton, OR, USA). The short arm of *Cd44* (3623 bp) was cut out from pCD44-KO vector with *Psp*OMI and *Nhe*I, and was ligated in pTVFlox-0 5́ (constructed by Dieter Riethmacher and Karin Gottschling) from the neo-cassette with *Not*I and *Xba*I to obtain the pTVFlox-s.a. vector. The long arm of *Cd44* (6624 bp) was cut out from the pCD44-KO vector with *Hpa*I and *Bam*HI, and inserted into pTVFlox-s.a. vector with *Hpa*I and *Bgl*II 3́ from the neo-cassette. The final construct abbreviated as pCD44-cKO was linearized by *Bsa*I and electroporated into 129S2 ES cells. Correctly targeted ES cells were identified, the neo-cassette removed using pCMV-*Cre* and positive clones injected into C57BL/6 blastocysts and transferred to 3H1 foster mothers. After germ-line transmission, *Cd44* floxed mice (*Cd44*^*fl/fl*^) were crossed to *K14Cre* mice (Daniel Metzger, Strasbourg, France) to result in *Cd44*^*Δker*^ mice. All experiments were performed with *Cd44*^*Δker*^ mice backcrossed to C57BL/6 mice for eight generations. Controls and mutants were identified by PCR genotyping on tail DNA using the following primers: *Cd44*^*−*^forward 5′-GGG AGC ATT GGC TGA CAA CA-3′, *Cd44*^*−*^reverse 5′-TTA CTC TGA TCA TGG CTC TC-3′, K14cre forward 5′-ATT TGC CTG CAT TACCGG TC-3′, K14cre reverse 5′-ATC AAC GTT TTG TTT TCG GA-3′.

*Cd44* germ-line knockout mice (*Cd44*^*−/−*^) with a C57Bl/6 background were a kind gift from Tak Mak (Toronto, Ontario, Canada). C57Bl/6J wild-type mice were obtained from Charles River Laboratory (Sulzfeld, Germany). Animals were housed and maintained under specific pathogen-free conditions in facilities approved by the Regierungspräsidium Karlsruhe. All animals were handled according to German regulations for animal experimentation. Experiments were authorized by the Regierungspräsidium (35–9185.81/G-110/10).

### Antibodies and reagents

The mouse CD44 pan antibody IM7 (BD Biosciences, Heidelberg, Germany, #553131) and the mouse CD44v6 antibody 9A4 have been described.^58^ The antibodies directed against Filaggrin (#PRB-417P), Keratin 6 (#PRB-169-P), Keratin 10 (#PRB-159P), Keratin 14 (#PRB.155P) and Loricrin (#PRB-145P) were purchased from Covance, HiSS Diagnostics (Freiburg, Germany). Isotype control antibodies mouse IgG (#sc-2025) or rat IgG (#sc-2026) were from Santa Cruz (Heidelberg, Germany), the secondary antibodies: goat anti-rabbit Alexa Fluor 488 (#A-11034), goat anti-rabbit Alexa Fluor 546 (#A-11071), goat anti-rat Alexa Fluor 488 (#A-11006) and goat anti-rat Alexa Fluor 546 (#A-11081) were purchased from Invitrogen (Karlsruhe, Germany). Secondary antibodies linked to horseradish peroxidase were from Dako (Hamburg, Germany, #P045001). The antibody against Ki67 was obtained from Abcam, Cambridge, UK (#Ab15580). 1-stearoyl-2-oleoyl-*sn*-glycero-3-phosphocholine (SOPC) and 1,2-dioleoyl-*sn*-glycero-phosphoethanolamine-3-N-(cap biotinyl; biotin-DOPE) were purchased from Avanti Polar Lipids (Alabaster, AL, USA), and neutravidin from Life Technologies (Darmstadt, Germany). Hydrazide-PEG_4_-Biotin and 1-ethyl-3-(3-diMEThylaminopropyl)carbodiimid were purchased from Thermo Fisher Scientific (Waltham, Massachusetts, USA).

### Primary keratinocyte culture

All experiments were exclusively performed with keratinocytes isolated from mouse tail skin epidermis. Mice were sacrificed by decapitation and the tail was removed, vertically cut with a razor and the skin was pulled off. The epidermis was separated from the dermis by placing the skin (dermal side down) into 1% trypsin for 1 h at 37 °C. The epidermis was cut into small pieces, transferred into Defined Keratinocyte-SFM (Serum Free Medium) (Life Technologies) containing 10% FCS and 0.25 mg/ml DNase1 and incubated 1 h at 37 °C. Disaggregated cells and tissue clumps were poured through a sterile cell strainer (70 *μ*m), centrifuged and re-suspended in Defined Keratinocyte-SFM medium, seeded on coated plates (Coating Matrix Kit, Life technologies) and incubated at 37 °C, 5% CO_2_. On the next day the cells were washed with PBS and suspended in new Keratinocyte-SFM medium. Cells were used after 2–5 days of culture.

### Preparation of genomic DNA from different tissues and from keratinocytes

Genomic DNA from skin, liver, kidney and lung were isolated as described.^59^ Keratinocytes were lysed in a buffer containing 50 mM Tris-HCl, pH 7.6, 1 mM EDTA, 100 mM NaCl, 0.2% SDS and proteinase K (100 *μ*g/ml) for 2 h at 60 °C. DNA was precipitated by addition of 100% ethanol followed by centrifugation for 2 min at 10 000 r.p.m. Pellets were washed with 70% ethanol, centrifuged for 2 min at 10 000 r.p.m., dried and suspended in TE buffer.

### Southern blot analysis

Genomic DNA (15 *μ*g) from mouse keratinocytes was digested with Hind III (Fermentas, St. Leon-Rot, Germany) and resolved on a 0.8% agarose gel. 0.4 M NaOH was used to transfer DNA fragments from the gel to a Zeta Probe GT Membrane (Bio-rad, Munich, Germany). The membrane was baked for 2 h at 80 °C. Hybridization was performed at 65 °C overnight using the Church buffer. A probe was produced via PCR from the Bacterial artificial chromosome RP23-408N14 (BACPAC Resources-CHORI, Oakland, CA, USA) using the primers Cd44^SB^ forward 5′-AAG CAC TTC ACT GGC TGA GC-3′, Cd44^SB^ reverse 5′-TCT GCA CAG TCA AGA CTC TGC-3′.

### RNA isolation and RT-PCR

Total RNA was isolated using Trizol (Sigma-Aldrich, Taufkirchen, Germany), complementary DNA was synthesized using Ready-To-Go-You-Prime-First-Strand Beads (GE Healthcare (VWR), Bruchsal, Germany) and RT-qPCR performed using GoTaq RT-qPCR Master Mix (Promega, Mannheim, Germany) and Eppendorf fluorescence thermocyclers (Eppendorf, Wesseling-Berzdorf, Germany), all following manufacturers' instructions. The 2^ΔΔCT^ method was used to quantify amplified fragments. Expression levels were normalized using at least one housekeeping gene (*rps29* and/or *rpl4*). The sequence of the qPCR primers can be found in ref. 47. The sequences of the primers used for CD44 are: 5′-GCACTGTGACTCATGGATCC-3′ present in exon 16 and 5′-GCACTGTGACTCATGGATCC-3′ in exons 17–18.

### Wound healing experiments

Ten to twelve-week-old male *Cd44*^*Δker*^ mice and control animals were anesthetized by intraperitoneal injection of ketamine (80 mg/kg body weight)/xylazine (10 mg/kg body weight). The dorsal hair was shaved and the exposed skin was cleaned with 70% ethanol. Full-thickness excisional skin wounds (usually two wounds) were performed on both sides of the dorsal middle line using a 4 mm biopsy-punch. The clot was removed daily and pictures of the wound area were taken. The wound area was calculated using the Image J Program (National Institutes of Health, Bethesda, MD, USA). The wound closure was expressed as percentage of recovery with respect to the initial wound area.

To specifically measure the contribution of keratinocytes, the wound healing experiment was performed without removing the clot. Animals were killed at indicated time points and the wounded tissue including the surrounding wound margin skin was harvested and embedded in Tissue Freezing Medium (Leica Microsystems, Wetzlar, Germany). Wound sections were stained immune fluorescently for the activation marker keratin 6 and the distance between the migration fronts of the keratinocytes was determined.

For each experiment six wounds per genotype were analyzed. Error bars represent the S.E.M. statistical analysis was performed by Student's *t* test. *P*<0.05 or *P*<0.005 is indicated by one and two asterisks respectively.

### TPA treatment

Seven-week-old female animals were shaved on the dorsal area of the skin and 48 h later TPA (5 nM/100 *μ*l acetone) or acetone alone was locally applied topically on the back skin. This treatment was performed two times every 48 h. 48 h after the last treatment, the skin was harvested and fixed in 4% paraformaldehyde overnight, processed in a tissue processor for dehydration and embedded in paraffin for histological analyses. Sections were stained using H&E or with different sets of keratin markers.

### Hair plucking

The hair was plucked on a 0.5 cm^2^ dorsal surface of 8-week-old female mice. 10 days later hair from the same area was plucked again and the treated region was harvested after 2 more days. Thereafter, the tissues were stained immune fluorescently for the proliferation marker Ki67. For quantification Ki67-positive cells were counted. As a control, skin samples were taken at a distance from the plucked surface.

### Colony formation assay

Murine keratinocytes of each genotype were seeded on coated (Coating Matrix mix, Thermo Fisher Scientific, Cat.nr. R-011-K) 6-well plates at 1000 cells/well. The cells were grown for 10 days. Plates were gently washed once with PBS and fixed with 3.7 % formalin for 10 min at RT. After fixation, plates were rinsed once again with PBS and colonies stained with 0,2 % crystal violet solution in 10 % ethanol for 15 min at RT. To remove the excess staining, the plates were rinsed several times with PBS. Colonies of more than 50 cells were counted under the microscope.

### Immunofluorescence and immunohistochemistry

For immunofluorescence analysis, frozen or paraffin sections of the skin were cut into 7 *μ*m-thick sections and mounted on glass slides. The unspecific binding of the antibody was blocked by 5% FCS in PBS for 1 h at room temperature followed by overnight incubation with primary antibodies diluted in blocking solution: IM7 (2 *μ*g/ml); 9A4 (2 *μ*g/ml); *α*filaggrin (1 *μ*g/ml); *α*keratin 6 (1 *μ*g/ml); *α*keratin 10 (1 *μ*g/ml); *α*keratin 14 (1 *μ*g/ml); *α*loricrin (1 *μ*g/ml). After 3 washes with PBS, the respective Alexa Fluor labeled secondary antibodies (1:500) were applied with DAPI (Sigma-Aldrich) in blocking solution for 1 h at room temperature, washed thee times with PBS and mounted.

Keratinocytes were fixed in 4% paraformaldehyde for 20 min at room temperature. After washing for three times with PBS cells were incubated with 5% FCS in PBS for 1 h at room temperature treated with IM7 (2,5 *μ*g/ml) over night, rinsed in PBS for 3 times, incubated with Alexa Flour labeled secondary antibody (1:500) for 1 h at room temperature, washed and then mounted using the Fluorescent Mounting Medium (Dako).

For H&E staining, paraffin sections were deparaffinized in xylene, rehydrated (100% EtOH 1 min, 95% EtOH 1 min, 80% EtOH 1 min, 70% EtOH 1 min, H_2_O), stained with hematoxylin for 1–5 min, rinsed in H_2_O and stained with eosin for 1 min. Thereafter, slides were dehydrated (95% EtOH 1 min, 100% EtOH 1 min, Xylene 5 min 2X) and mounted.

To detect HA by means of a biotinylated HA-binding proteins (Bio-HABPs, 2.5 *μ*g/ml, Merck, Darmstadt, Germany) cells were seeded in 12-well plates on sterile cover slips and starved for 24 h. Cells were fixed for 10 min with 4% paraformaldehyde (PFA) in PBS, then blocked with 5% FCS in PBS and afterwards incubated overnight with the biotinylated HA-binding protein. After incubation with Streptavidin/Alexa488 (0.4 *μ*g/ml Invitrogen) for 45 min, nuclei were stained with DAPI (Dako, Hamburg, Germany). For controls, cells were incubated with 50 U/ml Bovine Testis hyaluronidase (Sigma-Aldrich) before fixation.

### Functionalization of supported membranes with biotinylated HA oligomers

#### Preparation of biotinylated hyaluronic acid oligomers

HA oligomers (13–16 monomers, 0.334 mg/ml) in 0.1 M MES buffer at pH 4.5 (1.00 mg, 91 nmol) were incubated with a 10-fold molar excess of hydrazide-PEG_4_-Biotin (0.50 mg, 910 nmol) and 1-ethyl-3-(3-diMEThylaminopropyl)carbodiimid (0.17 mg, *n*=912 nmol) at pH 4.5 (addition of 1 mM HCl). The reaction mixture was stirred for 12 h in an end-to-end motion at 25 °C, transferred to a pre-wetted Slide-A-Lyzer dialysis cassette with a molecular weight cut-off of 2 kDa (Thermo Fisher Scientific, Waltham, MA, USA) and dialyzed exhaustingly against HBS buffer (10 mM Hepes, 150 mM NaCl, pH 7.5).

#### Preparation of HA functionalized supported membranes

Cell incubation chambers were prepared according to our previous protocol.^38^ Then, supported membranes consisting of 2 mol% biotin-DOPE in SOPC were deposited inside the cell incubation chambers by vesicle fusion.^60^ Subsequent functionalization of the supported membrane with neutravidin (10 nM) and oligo-HA (10 nM) were performed as described.^38^ As the anchor lipids biotin-DOPE are monomerly incorporated into the matrix lipids, the average lateral distance between lipid anchors *<d>* and thus oligomers can be estimated from the molar fraction *x* of lipid anchors by inserting the value of the lipid area of *A*_lipid_=65 Å^2^.


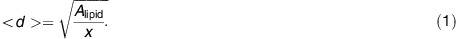


Excess HA oligomers were removed by rinsing with Keratinocyte-SFM medium. The measurement chambers were equilibrated at 37 °C before use for cell adhesion and migration experiments.

### Reflection interference contrast microscopy

Keratinocytes (5 × 10^4^ cells/cm^2^) were incubated for 5 h at 37 °C and 5% CO_2_ in cell incubation chambers in Keratinocyte-SFM medium. RICM was undertaken as described.^38^ Image corrections for shot noise and parabolic illumination profile were performed as described.^61^ The measured intensity was converted into heights, applying the RICM theory for finite INA and multiple reflecting layers, with refractive indices *n*=1.525 (glass substrate), *n*=1.486 (lipid membrane), *n*=1.335 (cell medium) and *n*=1.37 (cytosol).^62^ The adhesion area *A*_Adh_ was calculated from the average height and s.d. in each pixel. The fraction of adherent cells *χ* was determined by dividing the number of adherent cells, which were identified by RICM by pixel thresholding, by the total number of cells visible on phase contrast images.

### Statistical image analysis of stochastic morphological dynamics

Keratinocyte migration was followed in cell incubation chambers (5 × 10^4^ cells/cm^2^) in Keratinocyte-SFM medium by recording time-lapse movies over 12 h with a frame rate of 0.017 Hz with a Keyence BZ-9000 microscope (Keyence, Osaka, Japan), which was equipped with a Plan Fluor 40x/0.6 air objective. The movies were drift-corrected utilizing cross-correlation analysis. Morphological dynamics of migrating keratinocytes were quantified as described previously.^38^ The peripheral edge of each cell *r* in each phase contrast image of a time-lapse series of 60 min was detected by pixel contrast and plotted as a function of *Θ* for 0–360° in laboratory coordinates. An amplitude map *r(θ,t)* was obtained by displaying the time evolution of the radial distances *r*. Thereafter, the corresponding autocorrelation map *Γ*_*rr*_*(θ,t)* was calculated according to


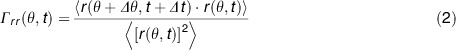


A Fourier transform of the radial distance in *Θ* and *t* resulted in the determination of different modes *m* of deformation.


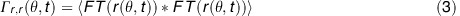


Finally, an integration over all modes *m* of deformation was performed to quantify the total power of cell deformation 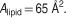


### Statistics

The underlying number of experiments per data point corresponds to three experiments. In summary, the fraction of adhered cells and the adhesion area *A*_Adh_ were calculated for a total number of 30 cells per data point, which were presented in the corresponding figures as means±s.d. Data characterizing the morphological dynamics of keratinocytes, comprise a total number of 20 cells per data point and represent mean±s.d. of the mean. In addition, a Mann–Withney test was applied to test for statistical significance.

### AFM elasticity measurements

AFM elasticity measurements were performed using a NanoWizard II AFM (JPK Instruments) mounted on top of an inverted optical microscope (Carl Zeiss AxioObserver A1). Indenter probes were prepared by gluing a single ∅10 *μ*m silica bead (Kisker Biotech) to the apex of a tipless V-shaped cantilever (NP-O, Veeco, Germany). Colloidal indenter probes were calibrated before and after each measurement. Frozen mouse skin tissue slices were attached to a microscopy slide and rehydrated in PBS for 20 min at room temperature. Subsequently, the indenter bead was positioned over dermal and epidermal regions as identified by light microscopy. Indentation measurements were performed by extending the closed-loop z-scanner with a constant rate of 1 *μ*m/s until reaching a pre-set force of 2 nN, typically corresponded to cell indentation depths of <1.5 *μ*m. Cell elasticity values were calculated from the obtained force-distance curves by applying a Hertzian mechanics model to the initial 500 nm of indentation. Elasticity measurements were performed on at least 15 different locations within the dermal or epidermal regions, using skin slices from at least three different animals per condition. The statistical significance between experimental groups was determined using a one-way ANOVA test.

## Abbreviations

HA: hyaluronan
EGF: epidermal growth factor
EGFR: epidermal growth factor receptor
HGF: hepatocyte growth factor
VEGF: vascular endothelial growth factor
INL: inner nuclear layer
FGF: fibroblast growth factor
FGFR: fibroblast growth factor receptor
TPA: 12-O-tetradecanoylphorbol-13-acetate
AFM: atomic force microscopy
ICAM-1: intercellular adhesion molecule 1
HABP: HA-binding protein
RICM: reflection interference contrast microscopy
