# Supplementary material for: Epidermal-specific deletion of CD44 reveals a function in keratinocytes in response to mechanical stress
Source: Cell Death Dis. 2016 Nov 10;7(11):e2461–. doi: 10.1038/cddis.2016.342 (PMC5260879; doi:10.1038/cddis.2016.342)
Supplement: Supplementary Information [file cddis2016342x1.docx]

**Supplementary material**

**Legends to supplementary figures:**

**Figure S1. Abrogation of CD44 expression in the epidermis and hair follicles. (A)** PCR strategy. Schematic representation of Lox-P sites and the primers in the *Cd44* floxed gene. Deletion of exon 3 in the epidermis leads to a 429bp DNA fragment while in other tissues the fragment is 3034bp. PCR analysis on cDNA obtained from the various organs of the *CD44^Δker^* and control mice. Cre and GAPDH are used as control. **(B)** Southern Blot analysis of cDNA from keratinocytes of *Cd44^Δker^* mice and control animals. Upon Hind III digestion a 2,7kB fragment will be generated upon removal of exon 3 whereas the fragment is 5,2kB if exon 3 is not deleted. (**C**) qRT-PCR for *Cd44* on keratinocytes isolated from control and *Cd44^Δker^* skin. Bars represent means +/- SD of technical duplicates relatives to control (set to 1). Of note, the primers for the PCR in Figure S1A monitor the expression of *Cd44* exon3 sequences, whereas the primers for the qPCR in Figure S1C monitor expression of the whole CD44 mRNA.

**Figure S2. Wound healing in *Cd44* germline knockout mice. (A)** Wound sections of Cd44 germline knockout mice or control animals were prepared at the indicated times and subjected to indirect immune fluorescence staining of keratin 6 (red). The nuclei were counterstained with DAPI (blue). Images were taken by fluorescence microscopy (KEYENCY BZ-9000) using a 2x objective. Bar = 500µm. The wound closure was quantified by measuring the distance between the keratinocyte migration fronts (shown with white arrows). Bars +/- SEM n=6. **(B)** qRT-PCR for the indicated differentiation markers and AP-1 genes using RNA isolated from control and *Cd44^Δker^* keratinocytes treated for the indicated periods with TPA. Bars represent means +/- SD of technical duplicates. Values are relatives to control at time zero (set to 1).

**Figure S3. (A) HA distribution in the epidermis.** HA staining (brown) was performed on PFA fixed and paraffin-embedded (FFPE) sections during homeostasis or wound healing of control and *Cd44^Δker^* mice using a biotinylated HA binding protein. The immunohistochemical staining was visualized using 0.05% 3,3’-diaminobenzidine. The specificity of the HA staining was proven by using human FFPE intestine tissue showing strong HA expression within the lamina propria of intestinal crypts (red arrow) as well as an exclusive delicate staining of stromal (reticular) cells within the germinal centers (B-cell areas) of Peyer’s patches (green arrows).

**(B) Supported membranes as a cell mimetic model system.** Lipid bilayer deposited on a glass substrate functionalized with HA oligomers at a defined average intermolecular distance of ligand molecules <d> ~ 6 nm served as *in vitro* model for skin tissue. Lipid bilayer on solid support without functionalization was utilized as control. **(C)** Morphological dynamics of keratinocytes on supported membranes. Phase contrast image with cell trajectory (left side), amplitude (middle part) and autocorrelation map (right side) for CD44 keratinocyte (control or *Cd44^Δker^*) on supported membranes displaying HA_oligo_ at <d> ~ 6 nm (upper part) or on supported membranes without functionalization (lower part). Scale bar =10 µm.
