# Supplementary figures and images for: Epidermal-specific deletion of CD44 reveals a function in keratinocytes in response to mechanical stress
Source: Cell Death Dis. 2016 Nov 10;7(11):e2461–. doi: 10.1038/cddis.2016.342 (PMC5260879; doi:10.1038/cddis.2016.342)

Supplementary Figure S1

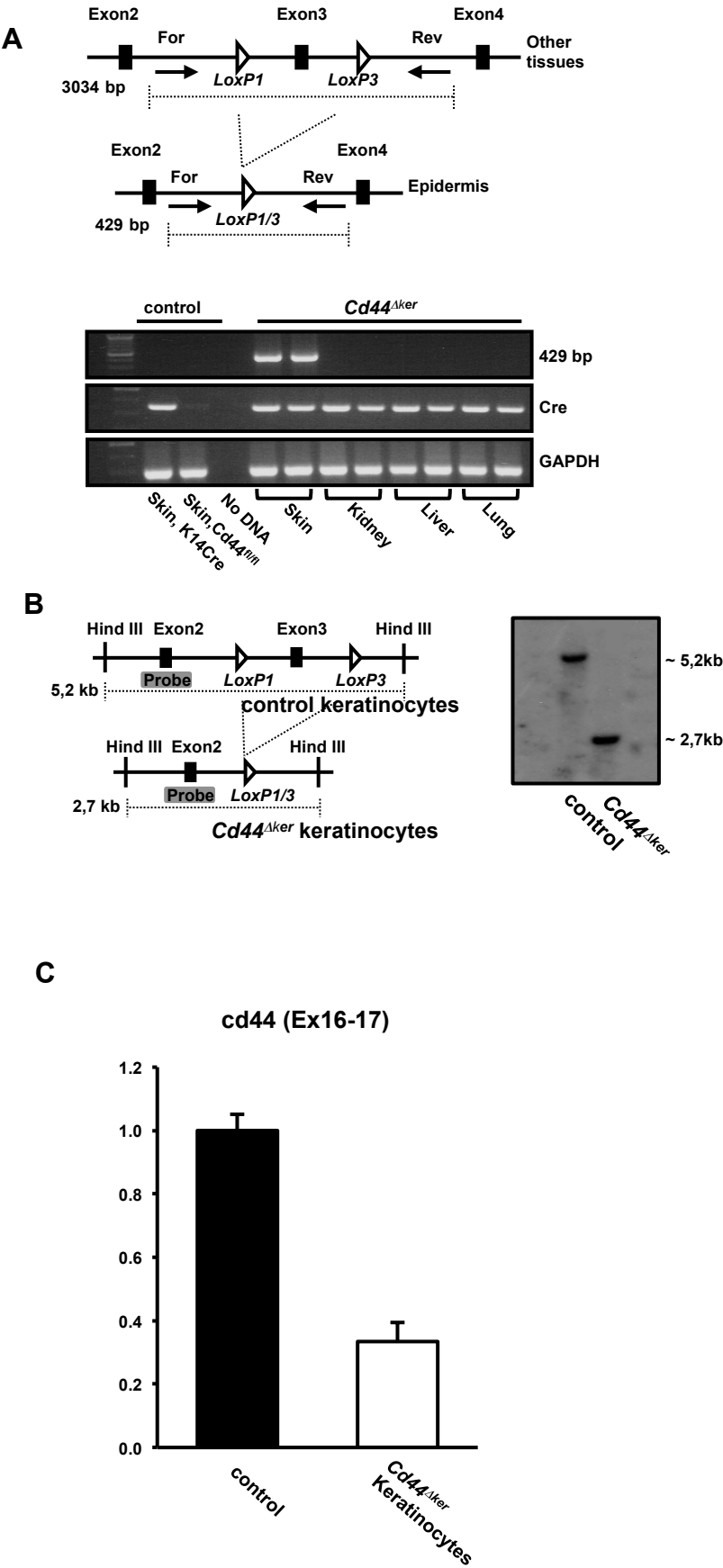

Supplement: Supplementary Figure S1 [file cddis2016342x2.pdf]

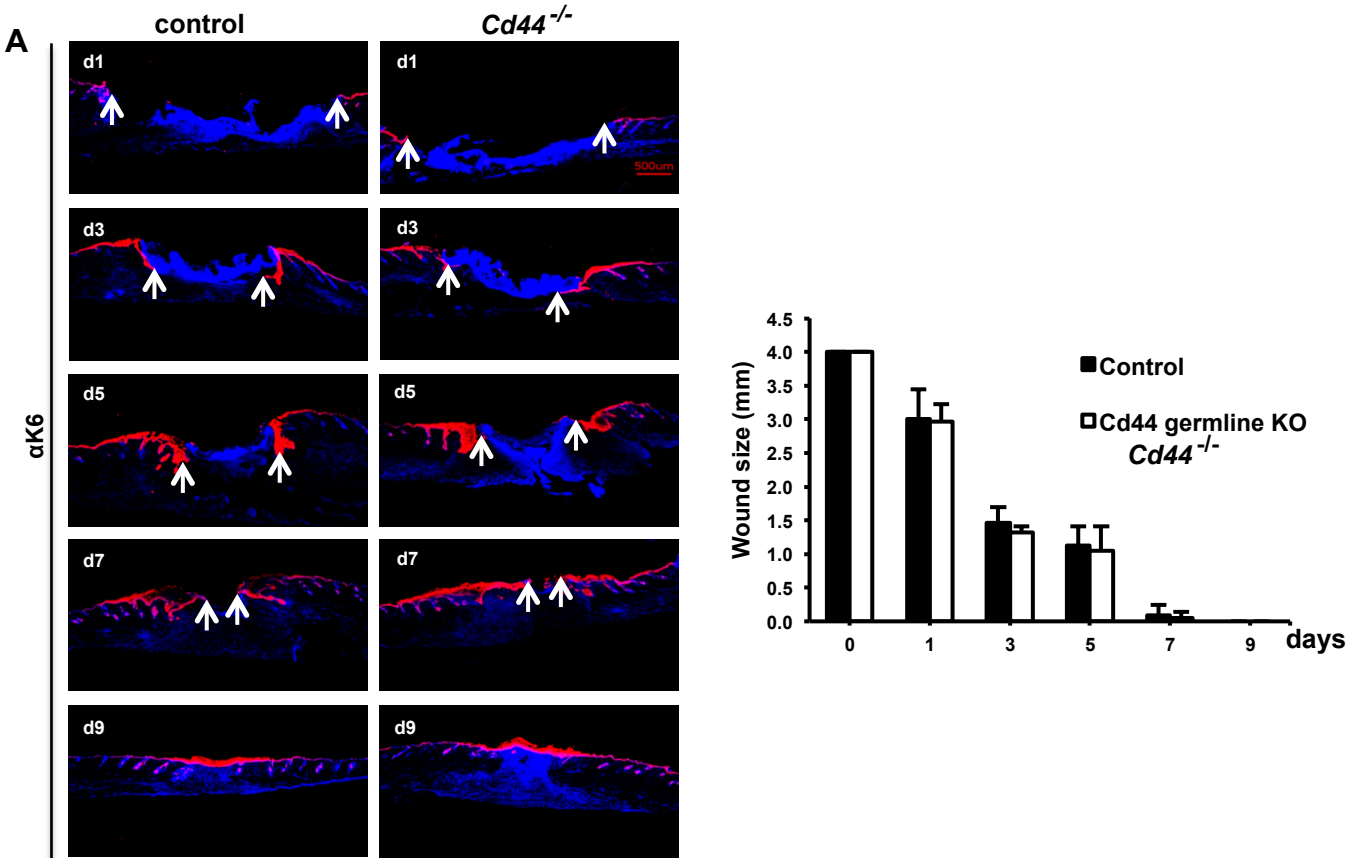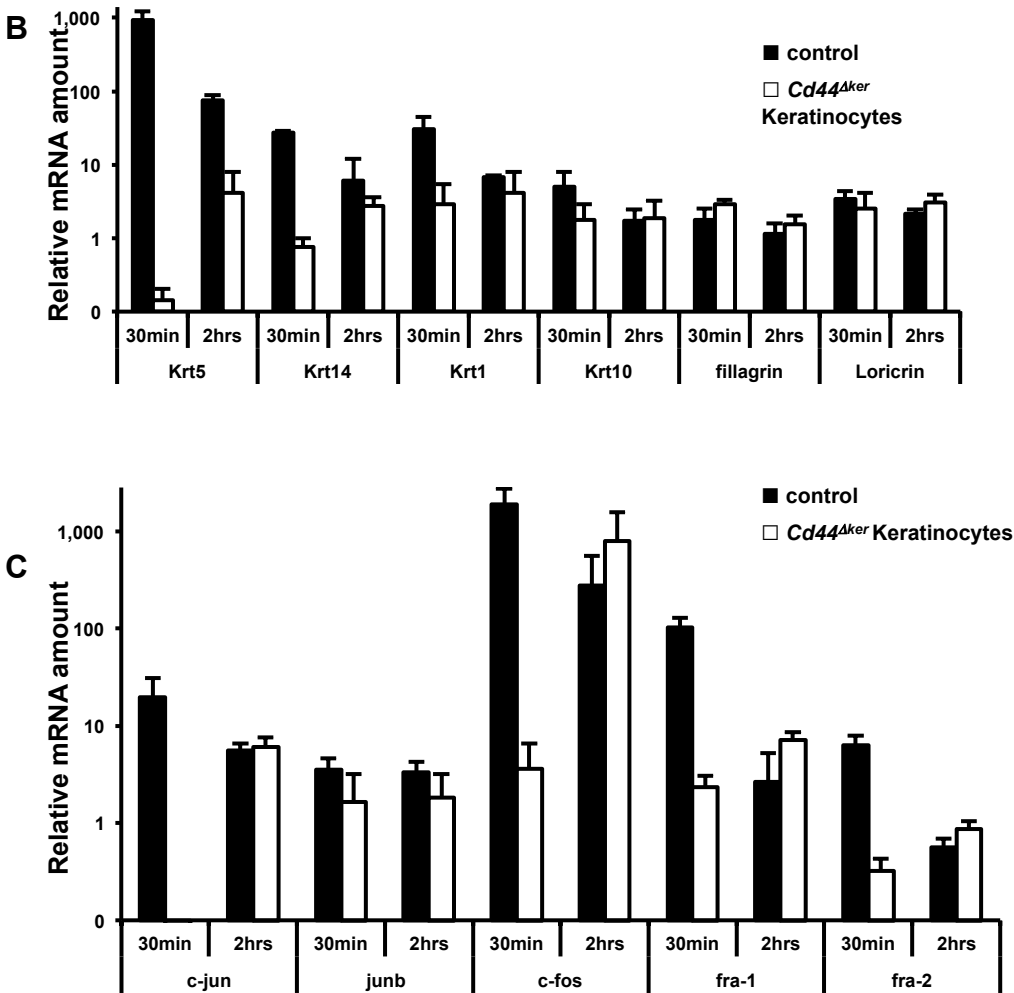

Supplement: Supplementary Figure S2 [file cddis2016342x3.pdf]

**A**

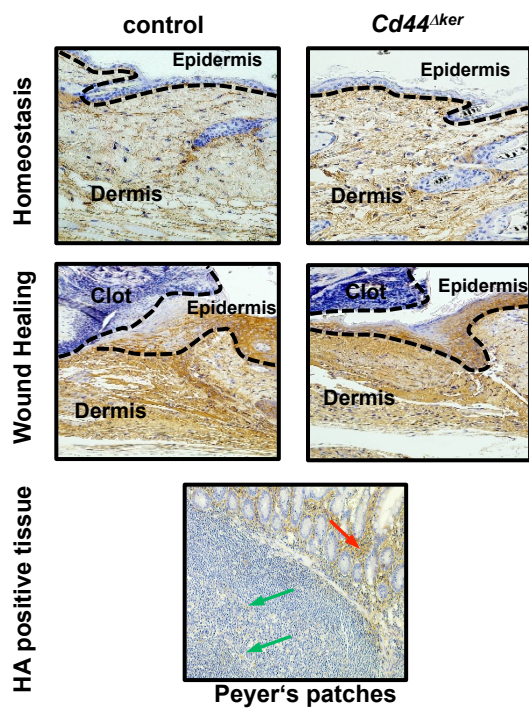

**B**

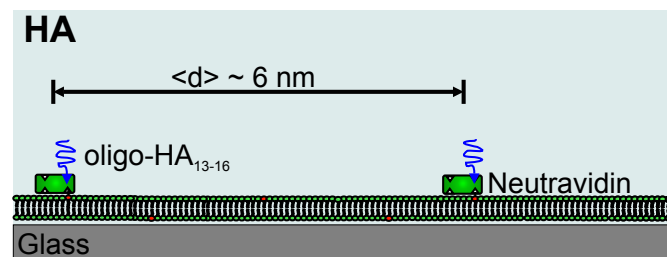

**C**

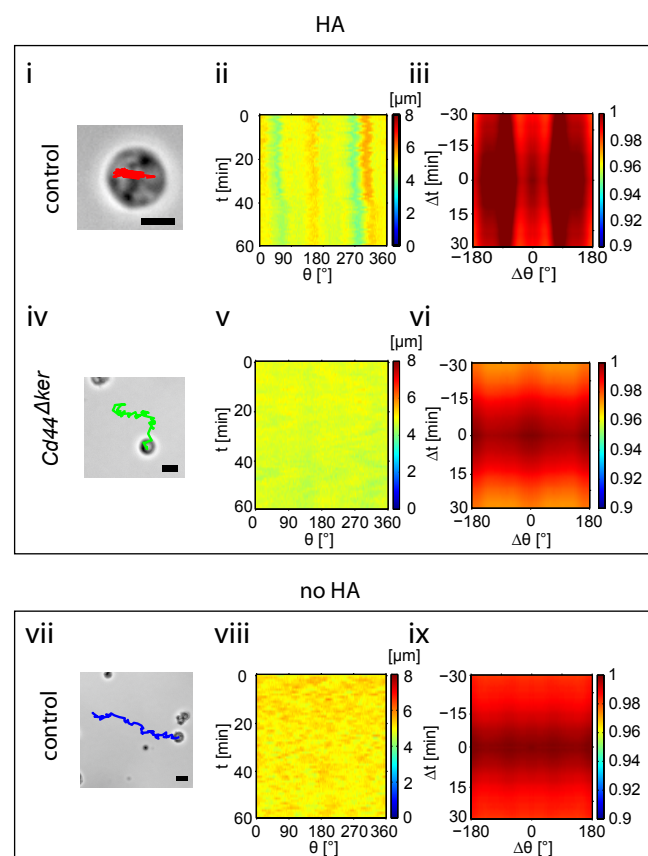

Supplement: Supplementary Figure S3 [file cddis2016342x4.pdf]
